# Pattern of macronutrients intake among type-2 diabetes mellitus (T2DM) patients in Malaysia

**DOI:** 10.1186/s40795-022-00648-y

**Published:** 2023-01-30

**Authors:** Md Isa Zaleha, Ismail Noor Hassim, Mohd Tamil Azmi, Jaafar Mohd Hasni, Ismail Rosnah, Mohamed Noor Khan Nor Ashikin, Mat Nasir Nafza, Ab Razak Nurul Hafza, Zainol Abidin Najihah, Yusof Khairul Hazdi

**Affiliations:** 1 Department of Community Health, Faculty of Medicine, UKM Medical Centre, Universiti Kebangsaan Malaysia, Kuala Lumpur, Cheras Malaysia; 2 Faculty of Medicine, Universiti Teknologi MARA Sungai Buloh, Sungai Buloh, Selangor Malaysia; 3 Department of Diagnostic & Allied Health Science, Faculty of Health and Life Sciences, Management & Science University, Shah Alam, Selangor Malaysia

**Keywords:** Type 2 diabetes mellitus (T2DM), Macronutrients, Adult, Obesity

## Abstract

**Background:**

The incidence of type 2 diabetes mellitus (T2DM) is rising rapidly in Malaysia. Modifying dietary intake is key to both the prevention and treatment of T2DM. This study aims to investigate the pattern of macronutrient intake among T2DM patients in Malaysia.

**Methods:**

This study was carried out on adults aged between 35 and 70 years, residing in urban and rural Malaysian communities. A series of standardised questionnaires was used to assess the sociodemographic information, dietary intake and physical activity level of 15,353 respondents who provided informed consent to participate in this study. Blood sampling (finger prick test) and physical examination were performed to obtain blood glucose and anthropometric data, respectively. The Chi-square test was used to assess differences in the trends of macronutrient intake among T2DM patients.

**Results:**

The total number of participants diagnosed with T2DM in this study was 2254. Of these, 453 (20.1%) were newly diagnosed, 1156 (51.3%) were diagnosed for ≤5 years and 645 (28.6%) were diagnosed for > 5 years. Male patients show that there were significant differences among the three groups of T2DM according to the following variables: age, BMI, residency, participant comorbidity of hypertension, family history of T2DM and hypertension, and active smoker. Meanwhile, female patients show significant differences among the three groups of T2DM according to the following variables: age, BMI, marital status, education level, residency, participant comorbidity of hypertension and family history of T2DM. Most of the male patients consumed appropriate proportions of carbohydrate (458, 60.7%) and protein (618, 81.9%). However, female patients did not show any significant differences of the macronutrients intake among the three groups of T2DM patients.

**Conclusions:**

The pattern of dietary intake among T2DM patients in this study showed consumption of carbohydrate and protein within the range of Malaysian RNI, coupled with high fat intake. Compliance with the Recommended Nutrient Intake (RNI) was satisfactory for both carbohydrate and protein but not for fat. The pattern indicated a preference for fat rather than protein when carbohydrate intake was restricted.

## Introduction

The global prevalence of diabetes mellitus is estimated to rise from 9.3% (463 million cases) in 2019 to 10.2% (578 million) by 2030 and 10.9% (700 million) by 2045 [1, 2]. The prevalence of T2DM has been escalating rapidly in developing countries such as Malaysia [3]. The National Health and Morbidity Surveys (NMHS) in Malaysia reported an increase in T2DM prevalence from 15.2% in 2011 to 18.3% in 2019 [4, 5]. Meanwhile, the NHMS also reported that the prevalence of newly diagnosed T2DM cases (amongst those not known to have T2DM) in Malaysia increased from 8.0% in 2011 to 8.9% in 2019 [4, 5]. This group has the same risk of developing complications of diabetes as already-diagnosed T2DM patients. Newly diagnosed T2DM patients are more prone to developing T2DM complications at a later stage due to uncontrolled glucose levels [6]. T2DM is associated with both unmodifiable factors (age and genetics) and modifiable risk factors (obesity and dietary intake) [1].

Modification of dietary intake is key to both the prevention and treatment of T2DM [7]. The Nutrition Division of the Ministry of Health, Malaysia, has been promoting healthy eating practices among the Malaysian population to facilitate a higher quality of life. The healthy eating campaign included advocacy for the food pyramid guideline and the recent healthy meal intake guide of ‘quarter quarter half’ plate portions [8]. These campaigns were derivatives of the macronutrient guideline provided by the Malaysian Recommended Nutrient Intake (RNI). The RNI suggested that the general macronutrient requirements for an adult were 50–65% of total energy intake (TEI) from carbohydrate, 10–20% of TEI from protein and the remaining 25–35% of TEI from fat [9]. The Malaysian Adult Nutrition Survey (MANS) has shown that total carbohydrate intake decreased from 59% of TEI in 2003 to 55% in 2014, while the total protein and fat intake increased from 14 to 16% and 27 to 29%, respectively, for the same years [10, 11].

In general, there is none equivocal guidelines regarding macronutrients intake among T2DM patients. Malaysian Ministry of Health (MOH) provides suggestion of macronutrients intake among T2DM patients as follows; 45–60% carbohydrate, 15–20% protein, and 25–35% fat of TEI [12]. This guideline was similar to Canadian Diabetes Association (CDA) that suggest distribution macronutrients intake byT2DM patients were 45–60% carbohydrate, 15–20% protein, and 20–35% fat of TEI [13]. However, both guidelines emphasise individualisation of the macronutrients’ distribution based on weight, glycaemic and other metabolic goals, cultural preferences and individual lifestyle [12, 13]. Instead of inconclusive evidence for the best macronutrient proportion for all patients with T2DM, Hamdy & Barakatun-Nisak concluded that lower carbohydrate consumption (< 40% from TEI), intake of low glycemic index food, and balancing macronutrients improve postprandial blood glucose levels [14]. Besides, dietary patterns such as the Mediterranean diet or Dietary Approaches to Stop Hypertension (DASH) diet, are beneficial in managing diabetes [14].

Although there was evidence showing dietary factors in the development and management of T2DM, studies regarding dietary pattern for diabetes prevention and management in Malaysia is still lacking. Studies have previously been conducted in different settings to assess the dietary intake of T2DM patients in Malaysia [15–17]. However, these studies did not address dietary intake among newly diagnosed T2DM patients. Therefore, this study aimed to investigate the pattern of macronutrient (carbohydrate, protein and fat) intake among Malaysian adults with newly diagnosed and already-diagnosed T2DM.

## Methodology

### Design of the prospective urban and rural epidemiological (PURE) study

The PURE study has been described in previous literature [18–20]. It is a large-scale, international study of the incidence, mortality and risk factors associated with non-communicable diseases, which includes individuals from urban and rural communities in 21 countries, including Malaysia. The PURE study is coordinated by the Population Health Research Institute (PHRI), Hamilton, Ontario, Canada. The Malaysian arm of the study is coordinated by two public universities, namely Universiti Kebangsaan Malaysia (UKM) and Universiti Teknologi MARA (UiTM). This study has enrolled 15,353 Malaysian individuals since 2007, and follow-up data collection is ongoing until 2030. Participants were recruited from selected urban and rural areas of Malaysia that represent the heterogeneity of the national population in terms of social and economic factors.

Participants were recruited based on purposive sampling. Sampling was executed by reaching out to the community leaders of the purposively selected study locations, which included all 14 states of Malaysia. Once agreement from the community leaders was obtained, a health screening program was implemented. Prior to recruitment, individuals from the community health screening program were asked about their interest in participating in the study. Interested individuals were followed up through home visits. Individuals aged between 35 and 70 years and living in the same household were also invited as study participants. Participants aged 35–70 were selected due to the prevalence of diabetes mellitus were high starting at the age of 35 years old compared to younger Malaysian population [4, 5, 21]. Meanwhile, elders aged above 70 years old were excluded due to various underlying health issues that could be confounding factor to this study. Only the household members intend to continue living in their current home for a further 4 years were selected to join this study to ensure the feasibility of long-term follow-up. Written informed consent was obtained after participants understood the provided study information and their rights as study participants.

To ensure standardised methods of data collection, research assistants were trained with comprehensive operation manuals, videos and workshops. Data were transferred electronically to the project office and coordinating centre at PHRI for quality control.

### Measurements

Participants were defined as having T2DM if they reported having been diagnosed with T2DM or had a glucose level of ≥7 mmol/L (fasting blood glucose) or ≥ 11.1 mmol/L (non-fasting blood glucose) [12]. The measurement of blood glucose was recorded as fasting blood sugar when the participants fasted for at least 8 hours prior to the test. The non-fasting blood sugar test was carried out for participants who did not fast prior to blood collection. The GlucoSure Plus blood glucose monitor was used to measure participants’ blood glucose levels. Participants were defined as newly diagnosed if they had never been diagnosed with T2DM but had an elevated blood glucose level at the baseline study. Those who were already diagnosed were stratified into either the diagnosed for ≤5 years or diagnosed for > 5 years group. The grouping was based on the number of years from diagnosis to the time of baseline data collection.

Participants’ habitual food intake was recorded using a validated food frequency questionnaire (FFQ) [22]. Participants reported the usual portion size of each food item in the FFQ and the average frequency of consumption. Then, macronutrients in terms of total energy, carbohydrate, protein and fat intake were calculated based on the Malaysian food composition and US Department of Agriculture food composition databases, with reference to nutrient databases containing the recipes of mixed dishes [23]. Participant dietary intake was stratified into three groups according to the RNI for Malaysians [9]. The recommended proportions of carbohydrate, protein and fat in the TEI were 50–65%, 10–20% and 25–30%, respectively [9].

Information on demographics, personal and family medical history, and active or passive smoking status was extracted from the validated PURE questionnaire and physical activity levels were assessed using the International Physical Activity Questionnaire (IPAQ) [19, 23, 24]. Demographic characteristics included age (rounded to the nearest year of birth), gender, race (Malay or non-Malay), marital status (single, married or divorced), education level (none, primary, secondary or tertiary) and employment status (yes or no). The residency area (urban or rural) was based on local government-gazetted areas. Rural areas were defined as areas occupied by less than 150 residents per square kilometre. Height and weight were measured using a portable stature meter and the TANITA (BC-558 Ironman®) segmental body composition analyser, respectively. Body mass index (BMI) was derived by dividing weight by height squared. BMI was categorised into underweight (< 18.5 kg/m^2^), normal (18.5–24.9 kg/m^2^), overweight (25–29 kg/m^2^) and obese (≥ 30 kg/m^2^).

The participants were asked whether they had been diagnosed with hypertension as comorbidity. Family medical history of T2DM and hypertension was defined as the occurrence of these comorbidities in at least one family member (father, mother or siblings) as reported by the participant. Active smoking status was categorised into current smokers and former tobacco smokers who had quit within the previous year, while inactive smoker was those who had never smoked. Passive smokers included those who had been exposed to environmental tobacco smoke at least once a week for the previous year. Physical activity level was considered low if it was < 600 Metabolic Equivalent (MET) min/week and high if it was ≥600 MET min/week [25, 26].

### Statistical analysis

The data were analysed using SPSS version 26. Chi-square test was used to assess differences in the T2DM groups stratified by gender according to the following variables: carbohydrate intake, protein intake, fat intake, age, BMI, race, marital status, education level, employment status, residency, participant comorbidities, family history of comorbidities, smoking status and physical activity level. The results were reported as frequencies and percentages, and a *p*-value of < 0.05 was considered significant.

## Results

A total of 15,352 participants completed the personal medical history assessment of T2DM. Of these, 2254 participants were diagnosed with T2DM without missing data on years of being diagnosed, age, gender and obesity. Finally, a total of 1719 participants completed the FFQs.

The total number of participants diagnosed with T2DM in this study was 2254. Of these, 453 (20.1%) were newly diagnosed, 1156 (51.3%) were diagnosed for ≤5 years and 645 (28.6%) were diagnosed for > 5 years. Table 1 shows the general characteristics of this study population stratified by gender. Among male, there were significant differences among the three groups of T2DM patients according to the following variables: age, BMI, residency, participant comorbidity of hypertension, family history of T2DM and hypertension, and active smoker. Majority of male participants that has been diagnosed with T2DM ≤5 years were aged 51–60 years old (207, 52.9%), overweight (314, 56.1%), resided in urban area (272, 53.6%), have comorbidity of hypertension (302, 57.4%), have family history of T2DM (228, 58.6%) and hypertension (201, 58.1%), and being active smokers (262, 55.3%). Meanwhile, female shows significant differences among the three groups of T2DM patients according to the following variables: age, BMI, marital status, education level, residency, participant comorbidity of hypertension and family history of T2DM. Most of the female participants that has been diagnosed with T2DM ≤5 years were aged 51–60 years old (253, 50.9%), overweight (350, 53.5%), married (513, 49.8%), have primary education level (275, 50.6%), resided in rural area (356, 52.1%), have comorbidity of hypertension (410, 56.6%) and have family history of T2DM (281, 55.8%).

**Table 1:** General characteristics of T2DM patients stratified by gender

	Male^#^ (*N* = 1004)				Female^#^ (*N* = 1250)			
	Newly diagnosed	Diagnosed ≤5 years	Diagnosed > 5 years	*p* value	Newly diagnosed	Diagnosed ≤5 years	Diagnosed > 5 years	*p* value
Demographic characteristics								
Age (year)				< 0.001*				< 0.001*
35–40	18 (35.3)	27 (52.9)	6 (11.8)		33 (40.7)	33 (40.7)	15 (18.5)	
41–50	54 (24.9)	122 (56.2)	41 (18.9)		99 (30.6)	170 (52.5)	55 (17.0)	
51–60	72 (18.4)	207 (52.9)	112 (28.6)		80 (16.1)	253 (50.9)	164 (33.0)	
61–70	52 (15.1)	163 (47.2)	130 (37.7)		45 (12.9)	181 (52.0)	122 (35.1)	
BMI				< 0.001*				< 0.001*
Underweight	5 (38.5)	4 (30.8)	4 (30.8)		8 (47.1)	5 (29.4)	4 (23.5)	
Normal	77 (32.6)	92 (39.0)	67 (28.4)		79 (30.7)	112 (43.6)	66 (25.7)	
Overweight	81 (14.5)	314 (56.1)	165 (29.5)		102 (15.6)	350 (53.5)	202 (30.9)	
Obese	27 (17.9)	85 (56.3)	39 (25.8)		49 (20.2)	129 (53.3)	64 (26.4)	
Race				0.530				0.138
Malay	182 (19.5)	485 (52.0)	265 (28.4)		241 (21.1)	584 (51.1)	318 (27.8)	
Non-Malay	12 (17.4)	33 (47.8)	24 (34.8)		16 (15.1)	52 (49.1)	38 (35.8)	
Marital Status				0.848				0.023*
Single	1 (9.1)	7 (63.6)	3 (27.3)		6 (26.1)	9 (39.1)	8 (34.8)	
Married	192 (19.8)	500 (51.4)	280 (28.8)		227 (22.0)	513 (49.8)	290 (28.2)	
Separated	3 (14.3)	12 (57.1)	6 (28.6)		24 (12.2)	114 (58.2)	58 (29.6)	
Education level				0.145				< 0.001*
None	12 (36.4)	14 (42.4)	7 (21.2)		29 (17.3)	93 (55.4)	46 (27.4)	
Primary	78 (19.3)	199 (49.3)	127 (31.4)		82 (15.1)	275 (50.6)	186 (34.3)	
Secondary	72 (17.7)	222 (54.5)	113 (27.8)		113 (26.3)	216 (50.3)	100 (23.3)	
Tertiary	34 (21.4)	83 (52.2)	42 (26.4)		33 (30.6)	52 (48.1)	23 (21.3)	
Employment Status				0.710				0.528
Yes	97 (20.1)	254 (52.6)	132 (27.3)		138 (21.4)	319 (49.5)	187 (29.0)	
No	96 (18.8)	264 (51.7)	151 (29.5)		116 (19.5)	313 (52.6)	166 (27.9)	
Residency				**0.002**				**0.002**
Urban	77 (15.2)	272 (53.6)	158 (31.2)		99 (17.5)	281 (49.6)	187 (33.0)	
Rural	119 (23.9)	247 (49.7)	131 (26.4)		158 (23.1)	356 (52.1)	169 (24.7)	
Participants’ comorbidities								
Hypertension				< 0.001*				< 0.001*
Yes	43 (8.2)	302 (57.4)	181 (34.4)		59 (8.1)	410 (56.6)	255 (35.2)	
No	153 (32.0)	217 (45.4)	108 (22.6)		198 (37.6)	227 (43.2)	101 (19.2)	
Family history comorbidities								
Diabetes				< 0.001*				< 0.001*
Yes	32 (8.2)	228 (58.6)	129 (33.2)		56 (11.1)	281 (55.8)	167 (33.1)	
No	163 (26.6)	289 (47.2)	160 (26.1)		201 (27.1)	353 (47.6)	188 (25.3)	
Hypertension				0.001*				0.169
Yes	46 (13.3)	201 (58.1)	99 (28.6)		92 (18.1)	270 (53.0)	147 (28.9)	
No	149 (22.8)	316 (48.3)	189 (28.9)		165 (22.4)	364 (49.4)	208 (28.2)	
Lifestyle								
Active smoker				0.024*				0.366
Yes	96 (20.3)	262 (55.3)	116 (24.5)		10 (29.4)	17 (50.0)	7 (20.6)	
No	100 (19.2)	254 (48.7)	168 (32.2)		247 (20.6)	607 (50.6)	345 (28.8)	
Passive smoker				0.117				0.083
Yes	58 (21.2)	145 (53.1)	70 (25.6)		96 (22.0)	232 (53.2)	108 (24.8)	
No	81 (17.6)	230 (49.9)	150 (32.5)		158 (20.3)	381 (48.9)	240 (30.8)	
Physical activity				0.484				0.928
Low	64 (17.8)	193 (53.8)	102 (28.4)		77 (21.7)	179 (50.4)	99 (27.9)	
High	119 (21.0)	289 (51.0)	159 (28.0)		167 (20.7)	413 (51.2)	227 (28.1)	

^#^data are shown as n (%)

*significant at *p*< 0.05

The Chi-square analysis showed that there were significant differences in carbohydrate and protein intake by male among the three groups of T2DM patients (Table 2). Most of the male patients consumed appropriate proportions of carbohydrate (458, 60.7%) and protein (618, 81.9%). However, female patients did not show any significant differences of the macronutrients intake among the three groups of T2DM patients.

**Table 2:** Proportion of carbohydrate intakes among T2DM patient

	Male^#^ (*N* = 755)				Female^#^ (*N* = 964)			
Dietary intake (% from TEI)	Newly diagnosed	Diagnosed ≤5 years	Diagnosed > 5 years	*p*-value	Newly diagnosed	Diagnosed ≤5 years	Diagnosed > 5 years	*p*-value
Carbohydrate intake				0.001*				0.432
< 50	49 (19.4)	131 (51.8)	73 (28.9)		60 (20.0)	160 (53.3)	80 (26.7)	
50–65	83 (18.1)	239 (52.2)	136 (29.7)		117 (19.9)	306 (52.0)	165 (28.1)	
> 65	20 (45.5)	16 (36.4)	8 (18.2)		22 (28.9)	34 (44.7)	20 (26.3)	
Protein intake				0.012*				0.352
< 10	5 (71.4)	2 (28.6)	0 (0.0)		3 (37.5)	5 (62.5)	0 (0.0)	
10–20	123 (19.9)	320 (51.8)	175 (28.3)		165 (21.0)	408 (51.8)	214 (27.2)	
> 20	24 (18.5)	64 (49.2)	42 (32.3)		31 (18.3)	87 (51.5)	51 (30.2)	
Fat intake				0.411				0.290
< 25	40 (25.2)	72 (45.3)	47 (29.6)		49 (22.9)	105 (49.1)	60 (28.0)	
25–30	48 (18.5)	136 (52.5)	75 (29.0)		58 (17.8)	168 (51.5)	100 (30.7)	
> 30	64 (19.0)	178 (52.8)	95 (28.2)		92 (21.7)	227 (53.5)	105 (24.8)	

Data are shown as n (%)

*significant at *p*< 0.05

## Discussion

### Basic characteristics of T2DM patients

A majority of the newly diagnosed T2DM patients were between 51 and 60 years old (72, 15.4%) among male patients and 41–50 years old (99, 30.6%) among female patients. The findings for male patients were comparable with those of the NHMS, which reported that the prevalence of newly diagnosed T2DM was highest among 50–59 years old Malaysian male (143, 47.4%) [5]. While for female, the NHMS has reported that the highest prevalence of newly diagnosed T2DM were also 50–59 years old (160, 52.6%) which is older compared to this study that show the highest prevalence among 41–50 years old [5].

In the present study, patients with overweight BMI were most common among those who were diagnosed for ≤5 years for male (314, 56.1%) and female (350, 53.5%). Similar trend were observed for obese patients with percentage of 56.3 and 53.3% among male and female patients respectively. A study done by Mafauzy et al. reported that 72% of T2DM patients were obese, which reflects the imbalance between energy intake and expenditure [27–29]. According to a Malaysian report, obese persons were recommended to reduce their initial weight by 5–10% over a period of 6 months [29]. To achieve this goal, medical nutrition therapy (MNT) was provided via individualised nutritional recommendations for T2DM patients with obesity [29]. Although overweight and obesity are well-known risk factors for type 2 diabetes, the disease also noticeable among newly diagnosed T2DM patients with normal BMI in this study (male: 77, 32.6%; female: 79, 30.7%). According to Gujral et al., diabetes development among patients with BMI < 25 kg/m^2^ might be due to impairments in insulin secretion, in utero undernutrition, and epigenetic alterations to the genome [30].

Our results showed that patients already diagnosed with T2DM for ≤5 years had a notably high prevalence of hypertension comorbidities (male: 302, 57.4%; female: 410, 56.6%), compared to newly diagnosed patients (male: 43, 8.2%; female: 59, 8.1%). Increased comorbidities would increase the risk of complications such as cardiovascular disease and impact the management of comorbidities, long-term survival and the health care system [6]. Other than that, family history of diabetes also shows similar trends. This is because comorbidities are heritable [31]. The results of the present study also showed that active smoking status among male participants was significantly related to T2DM. Smoking behaviours have been reported as risk factors contributing to T2DM [32].

### Pattern of macronutrient intake among T2DM patients

The Chi-square analysis showed significant differences among the three groups of T2DM patients in terms of carbohydrate and protein intake by male patients. Compliance with the recommended carbohydrate intake (50–65% of TEI) among the newly diagnosed, ≤ 5 years and > 5 years groups was 83 (18.1%), 239 (52.2%) and 136 (29.7%), respectively. Similarly, carbohydrate consumption less than recommended intake (< 50% of TEI) among the newly diagnosed, ≤ 5 years and > 5 years groups was 49 (19.4%), 131 (51.8%) and 73 (28.9%), respectively. Meanwhile, T2DM patients who consumed carbohydrate at proportions > 65% of TEI was highest among the group newly diagnosed (20, 45.5%), compared to ≤5 years (16, 36.4%) and > 5 years groups (8, 18.2%).

Compliance with the recommended protein intake (10–20% of TEI) among the newly diagnosed, ≤ 5 years and > 5 years groups was 123 (19.9%), 320 (51.8%) and 175 (28.3%), respectively. Similar patterns were observed for a protein intake of > 20% of TEI among the newly diagnosed, ≤ 5 years and > 5 years groups (18.5, 49.2 and 32.3%, respectively). In addition, very few T2DM patients consumed protein in amounts less than the recommended proportions. Although the differences were not significant, the majority of the T2DM patients consumed amounts of fat higher than the recommended proportion (> 30% of TEI).

A previous study conducted at the outpatient clinic of the University of Malaya Medical Centre reported that the mean proportions of carbohydrate, protein and fat consumed by T2DM patients were 56.9, 14.7 and 28.4% of TEI, respectively [15]. Another study by Chin et al. found that the mean proportions of carbohydrate, protein and fat consumed by T2DM patients were 60.0, 16.0 and 24.0% of TEI, respectively [17]. Meanwhile, this study shows that the mean proportions of carbohydrate, protein and fat intake among T2DM patients were 51.9, 17.7 and 30.4% of TEI, respectively (results were not shown). The present study found that the mean carbohydrate intake among T2DM patients was lower than in previous studies (51.9% vs 56.9 and 60.0% of TEI) [15, 17]. Conversely, the mean intake of protein (17.7% of TEI) and fat (30.4% of TEI) among T2DM patients in this study was higher compared to the previous studies [15, 17]. The mean proportions of macronutrients consumed by participants in this study were found to be within the range recommended in the clinical practice guidelines for the nutritional management of T2DM patients [12].

Overall, the results of this study showed that T2DM patients mainly consumed carbohydrate and protein within the range of recommended nutrient intakes (RNI) for Malaysia but had a high fat intake (Table 2). This pattern contradicted a review by Hussein et al., which concluded that Malaysian diabetics were more prone to consuming high amounts of carbohydrate and fat [29]. The differences may be because the former study included only already known T2DM patients, while this study included both newly diagnosed and already-diagnosed T2DM categories. Several studies have highlighted that the general dietary intake recommendations based on macronutrients were not easily followed by both the general population and T2DM patients [7, 14, 33, 34]. Furthermore, previous reviews have stated that the effectiveness of the existing guidelines, which set goals based on macronutrient quantity, was still equivocal in efforts to reduce the risk of T2DM [33, 34]. Thus, the Malaysian Ministry of Health (MOH) has been implementing MNT to provide individualised nutritional recommendations based on personal preferences to manage the dietary intake of T2DM patients [31]. However, this approach only showed a 16.4% rate of compliance among Malaysian T2DM patients despite its effectiveness in glycaemic control [31]. Despite the efforts of the MOH to manage T2DM, lack of patient compliance with dietary counselling remains a huge challenge for both health practitioners and T2DM patients themselves.

The main limitation of this study was the cross-sectional study design that only included baseline data. The causal and temporal effects of macronutrient intake on T2DM patients were not considered. Future research should include controlled trials or prospective data analyses so that the causal effects of specific components of carbohydrate, protein and fat can be studied.

## Conclusion

The T2DM patients in this study mainly consumed amounts of carbohydrate and protein within the range of RNI for Malaysia but had a high fat intake. Compliance with RNI recommendations for macronutrient proportions was satisfactory for carbohydrate and protein but not for fat. This trend shows that T2DM patients preferred fat over protein to replace lower proportions of carbohydrate. Further research regarding specific components of carbohydrate, protein and fat is necessary to understand the effects of macronutrients on T2DM in Malaysia. This study has also revealed a high proportion of newly diagnosed T2DM patients (20.1%), indicating a lack of awareness among the general population regarding this disease.

## Data Availability

The data that support the findings of this study are available from PHRI but restrictions apply to the availability of the data, which were used under license for the current study, and are not publicly available. Data are however available from the authors upon reasonable request and with permission from PHRI.

## Abbreviations

T2DM: Type-2 Diabetes Mellitus
TEI: Total energy intake
NHMS: National Health and Morbidity Survey
PURE: Prospective Urban and Rural Epidemiological Study
RESTU: Risk Epidemiological Study
FFQs: Food frequency questionnaires
TMC: The Malaysian Cohort
MOH: Malaysian Ministry of Health
MNT: Medical nutrition therapy
