# Correction: Pattern of macronutrients intake among type-2 diabetes mellitus (T2DM) patients in Malaysia

**DOI:** 10.1186/s40795-023-00687-z

**Published:** 2023-02-07

**Authors:** Zaleha Md Isa, Noor Hassim Ismail, Azmi Mohd Tamil, Mohd Hasni Jaafar, Rosnah Ismail, Nor Ashikin Mohamed Noor Khan, Nafiza Mat Nasir, Nurul Hafiza Ab Razak, Najihah Zainol Abidin, Khairul Hazdi Yusof

**Affiliations:** 1 Department of Community Health, Faculty of Medicine, UKM Medical Centre, Universiti Kebangsaan Malaysia, Kuala Lumpur, Cheras Malaysia; 2 Faculty of Medicine, Universiti Teknologi MARA Sungai Buloh, Sungai Buloh, Selangor Malaysia; 3 Department of Diagnostic & Allied Health Science, Faculty of Health and Life Sciences, Management & Science University, Shah Alam, Selangor Malaysia

**Correction: BMC Nutr 9, 21 (2023)**

**https://doi.org/10.1186/s40795-022-00648-y**

Following publication of the original article [1], the authors identified tagging errors in the author names:

Md Isa *(family name)* Zaleha *(Given Name)*

Ismail *(family name)* Noor Hassim *(Given Name)*

Mohd Tamil *(family name)* Azmi *(Given Name)*

Jaafar *(family name)* Mohd Hasni *(Given Name)*

Ismail *(family name)* Rosnah *(Given Name)*

Mohamed Noor Khan *(family name)* Nor Ashikin *(Given Name)*

Mat Nasir *(family name)* Nafiza *(Given Name)*

Ab Razak *(family name)* Nurul Hafiza *(Given Name)*

Zainol Abidin *(family name)* Najihah *(Given Name)*

Yusof *(family name)* Khairul Hazdi *(Given Name)*

The author group has been updated above and the original article [1] has been corrected.
